# Person-centred, integrated and pro-active care for multi-morbid elderly with advanced care needs: a propensity score-matched controlled trial

**DOI:** 10.1186/s12913-019-4397-2

**Published:** 2019-10-03

**Authors:** G. K. R. Berntsen, M. Dalbakk, J. S. Hurley, T. Bergmo, B. Solbakken, L. Spansvoll, J. G. Bellika, S. O. Skrøvseth, T. Brattland, M. Rumpsfeld

**Affiliations:** 1 Norwegian Center for e-health research, University Hospital of North Norway Trust, Universitetssykehuset, PB 35, 9038 Tromsø, Norway; 2 Clinic of general medicine, University Hospital of North Norway Trust, Universitetssykehuset, PB 100, 9038 Tromsø, Norway; 3 Clinic of general medicine, University Hospital of North Norway Trust, Harstad hospital, PB 1065, 9480 Harstad, Norway; 4 Director of Health and Care, Tromsø Municipality, PB 6900, Tromsø, 9299 Norway; 5 Institute of community medicine, UiT The Arctic University of Norway, PO Box 6050 Langnes, N-9037 Tromsø, Norway

**Keywords:** Person-centred care, Integrated care, Proactive care, Health care utilisation, Mortality, Propensity score matched controls

## Abstract

**Background:**

Person-centred care (PCC) focusing on personalised goals and care plans derived from “What matters to you?” has an impact on single disease outcomes, but studies on multi-morbid elderly are lacking. Furthermore, the combination of PCC, Integrated Care (IC) and Pro-active care are widely recognised as desirable for multi-morbid elderly, yet previous studies focus on single components only, leaving synergies unexplored. The effect of a synergistic intervention, which implements 1) Person-centred goal-oriented care driven by “What matters to you?” with 2) IC and 3) pro-active care is unknown.

**Methods:**

Inspired by theoretical foundations, complexity science, previous health service research and a patient-driven evaluation of care quality, we designed the Patient-Centred Team (PACT) intervention across primary and secondary care. The PACT team collaborate with the patient to make and deliver a person-centred, integrated and proactive multi-morbidity care-plan. The control group receives conventional care. The study design is a pragmatic six months prospective, controlled clinical trial based on hospital electronic health record data of 439 multi-morbid frail elderly at risk for emergency (re) admissions referred to PACT and 779 propensity score matched controls in Norway, 2014–2016. Outcomes are emergency admissions, the sum of emergency inpatient bed days, 30-day readmissions, planned and emergency outpatient visits and mortality at three and six months follow-up.

**Results:**

The Rate Ratios (RR) for emergency admissions was 0,9 (95%CI: 0,82-0,99), for sum of emergency bed days 0,68 (95%CI:0,52-0,79) and for 30-days emergency readmissions 0,72 (95%CI: 0,41-1,24). RRs were 2,3 (95%CI: 2,02-2,55) and 0,9 (95%CI: 0,68-1,20) for planned and emergency outpatient visits respectively. The RR for death at 3 months was 0,39 (95% CI: 0,22-0,70) and 0,57 (95% CI: 0,34-0,94) at 6 months.

**Conclusion:**

Compared with propensity score matched controls, the care process of frail multi-morbid elderly who received the PACT intervention had a reduced risk of high-level emergency care, increased use of low-level planned care, and substantially reduced mortality risk. Further study of process differences between groups is warranted to understand the genesis of these results better.

**Trial registration:**

ClinicalTrials.gov (identifier: NCT02541474), registered Sept 2015.

**Electronic supplementary material:**

The online version of this article (10.1186/s12913-019-4397-2) contains supplementary material, which is available to authorized users.

## Background

A rising number of elderly citizens heralds an increased number of persons with complex long-term needs (CLNs) who need and expect more of our care systems. Persons with CLNs typically face multiple care providers, organisations and specialists [1], and are especially vulnerable to care fragmentation [1–4]. This group also dominates the 5–10% top spenders who account for 2/3 of high-level health care spending both in Norway and internationally [5–7]. The expected increase in persons with CLN is threatening the sustainability of our healthcare systems [8]. Current care systems, designed for acute short-term needs, are struggling to meet the rising tide of persons with CLNs.

An extensive body of research indicates that critical elements of high-quality care for persons with CLNs include strong primary care with an inherent whole person, integrated and pro-active focus [9–14]. For simplicity, we will nickname this three-part solution PIP care, for Person-centred care (PCC), Integrated Care (IC) and Proactive care (Pro-C). There is an abundance of generally promising reviews regarding each of these three elements [15–24]. Yet, outcome evaluations remain unpredictable or weak with a high proportion of negative studies [15–22]. Reviewers report that there is little conceptual clarity on how to operationalise the PIP-elements, and for whom and in which contexts they will work [16, 17, 25]. We propose that the PACT study differs from previous research in two regards:

### A synergistic approach

Even though international recommendations highlight the complex and multi-faceted synergies between these three PIP-elements, studies in this field have generally followed the traditional approaches of the “rational-linear” approach, “… *When designing intervention and implementation strategies, as well as when conducting rigorous evaluations, there is a tendency to reduce messy real world situations into the individual component parts in an attempt to determine the relationships between them*” [20, 26]. However, to achieve better outcomes for persons with CLNs, the whole chain of care needs to change. We propose that the PIP-components are inter-dependent, and will only produce the expected effects if all the components are in place. The effect of an intervention where the PIP elements work synergistically together is mostly unexplored.

### A sharper definition of PCC

PCC is in many ways the foundation of PIP-care. PCC is widely recognised as desirable [13, 15, 27] yet the gap between the rhetoric and reality of PCC remains uncomfortably wide [28, 29]. The last few years, a sharper vision of PCC described by Bisognano as “flipped healthcare” is emerging [15, 30, 31]. It flips care focus from: What is the matter?" to “What matters to you?” It forces attention from professionally defined diagnosis and functional goals to personal goals. Here, we prefer the term “Goal-oriented Person-centred care” (Goal-PCC), to underline that in “flipped care,” the entire chain of care, including care planning, delivery, and evaluation is co-created with the person and driven by personal goals [32]. While Coulter found in her Cochrane review that Goal-PCC had a small but significant impact on outcomes in single disease contexts, neither Coulter nor we have found any studies that applied this concept to multi-morbid patients [15].

Norway has a robust primary care system [33], with high accessibility and low out of pocket expenses, among the highest life expectancies in the world, and excellent health outcomes in international comparisons, and a commitment to quality improvement for persons with CLNs [34–39]. However, persons with CLNs in Norway report the same challenges as other high-income countries, making the Norwegian context internationally relevant [39–41].

We designed the PACT intervention to address quality challenges identified by patients with CLNs in Norway [41], in alignment with the theoretical and empirical foundations of chronic care ideals. Central ideals were the Chronic Care Model [42], Goal-oriented Person-centred care [15, 31, 41, 43, 44], Shared Decision Making [45], integrated care pathways [46] and pro-active risk management [23, 47]. We were mindful of the complex nature of both the health service and the PACT intervention [48–50]. The care process for each person, here denoted as the individualised Patient Pathway (iPP) [41], is a product of a complex adaptive system (CAS) [49]. Key elements to support problem-solving in a CAS are: A good enough vision or goal, simple guiding rules and a wide space for innovation and creativity to reach common goals [49]. The goals and rules were set out in our previous work on the Person-centred integrated care quality framework [41]. Briefly, the iPP serves patient-defined goals [31, 44], with attention to PCC, IC and Pro-C, while incorporating insights and feedback from both the patient and other contributors is an ongoing attribute of the care process [48]. To the best of our knowledge, we have not found other studies on the effects of a complex, synergistic and “flipped” Goal-PCC model, which builds all three PIP-elements into the care model the way our Patient-Centred Team (PACT) intervention does.

### Aim

The PACT study aims to improve the triple aim of patient care experience, health outcomes and cost-benefit ratios for persons with CLNs. Due to delays in the collection of patient reported outcomes (see details later), we here report on early and available health outcomes using routine data from the electronic health record (EHR). We expect that the PACT intervention applied to a population of high risk frail multi-morbid elderly will stabilize the patient’s health situation through improved quality of care. As a result, we expect to see a reduced use of high-level emergency care and a higher use of low-level planned care. Hypothetically, use of emergency care could be reduced simply by denying patients adequate care. This would of course be unethical, and in this frail group, we would expect increased mortality. We therefore needed to show that reductions in the use of high-level emergency care was not accompanied by increased mortality. To summarize, the hypotheses (H) examined in this paper are that the PACT intervention will cause:

- H1: Reduced use of high-level emergency care .
- H2: Increased use of low-level planned care
- H3: Unchanged mortality risk

## Methods

### Study design, reporting and protocol changes

This study is a prospective propensity score (PS) matched controlled trial evaluating the effectiveness of the PACT service. Briefly, the use of routine data from the EHR, and the propensity score techniques described by Rubin [51–53], allowed us to create a valid comparison group even when the target population was defined by a discretionary referral process. The same EHR data also contained relevant process and outcome measures, which enabled a comparison of intervention and usual care. We follow recommendations for evaluation of complex interventions [54], complexity science [55] and quality improvement methodology [56].

The study design, which has been called a synthetic RCT, lies somewhere between the randomized controlled trial (RCT) and the observational study. We adhere to the CONSORT [57] STROBE [58], TIDieR [59] and the SAMPL [60] checklists for reporting on RCT, observational, intervention and biostatistical studies respectively.

We have previously published our study protocol at ClinicalTrials.gov (identifier: NCT02541474) and as a protocol paper [61]. We found it necessary to make the following protocol changes: 1) Due to delays in data collection, we report on secondary health outcomes before we are able to report on the primary outcome: the patient reported health experience by the SF12 questionnaire [62]. 2) Due to a high risk of healthy selection bias, we were allowed to include routine EHR data on all patients receiving PACT without their consent, while the original protocol planned inclusion based on written informed consent.

### The arguments for these two changes are

Patients, who had consented to receive care (care consent) from the PACT team, were eligible to be invited to give their informed written consent to participate in the PACT study (study consent). However, a high proportion suffered from acute serious or overwhelming morbidity at baseline. It is unethical to ask patients to fill a study consent or questionnaire in a situation where treatment and adjustment should take precedence. When patients had recovered sufficiently for study recruitment to be ethical, many were already past the 4 week study recruitment window and were no longer eligible. 2) A high proportion of persons with cognitive impairment were unable to provide informed consent and we found that family members were frequently uncomfortable answering personal questions on the patient’s behalf. For these two reasons, the recruitment and collection of the primary outcome based on patient reported data is delayed, is still ongoing and will continue to March 2019.

As early results on secondary health outcomes were important for health service decision makers regarding the funding of the PACT service, we looked for other ways to evaluate the PACT service. We also feared that a study based on informed consent would be flawed by a healthy selection bias. With this background, we sought and were granted permission (see ethics section) to extract routinely collected data from the EHR which contains both exposure and outcomes variables for both the intervention and control population, without asking for patient consent. This would ensure fair recruitment, irrespective of challenges to provide consent. The main ethical concern of this approach was to maintain privacy of the routine data provided to the research team. The privacy protection officer approved the project with the following precautions to ensure the privacy of the participants: We prepared pseudonymised analysis files, which means that direct identifiers such as names, addresses or id-numbers, were replaced with a pseudonym. We saved and used analysis files exclusively on “offline” devices/ computers. Only 3 researchers (GB, JH, TB1) had access to the analysis files. All intermediate and final analysis results are fully anonymous.

We, are therefore able to report here on the outcomes available to us from the EHR: healthcare utilisation and risk of death.

### The intervention design

The intervention was tailored to each patient, as long as the PACT team adhered to the following structures and principles.

#### The PACT structures

The PACT team is a cross-organisational (hospital and municipality) multi-disciplinary geriatric team set in two separate municipalities (M1 and M2), both with more than 20,000 inhabitants in Norway. Both M1 and M2 provide typical primary care services, and both host a local hospital. The local PACT team includes nurse coordinators, physio- and occupational therapists, geriatric nurses, pharmacists, medical secretaries and a medical doctor. The hospital and municipalities share financial, management and employment responsibilities for the team members. The PACT board represented significant stakeholders: Patients, primary and secondary care, research and health technology.

#### PACT care process

We designed PACT in a heuristic iterative process cycling between literature reviews [31], listening to and addressing patient [41], professional and manager experiences. PACT has many of the same components as other interventions directed at frail elderly persons: PACT is person-centred, applies a comprehensive geriatric assessment methodology to create an evidence-based care plan. PACT applies a combination of a case manager and a multi-professional team to address complex patient issues in line with international guidelines [11, 12]. However, PACT differs from the bulk of integrated care research in two critical respects:

1. The vision of a Goal-oriented PCC: The heart of the PACT intervention is a continuous process of trust-building, sensitive exploration and co-creation between professional and patient, aimed at capturing “What matters to you?”. Together with the person, PACT translates “What matters” into relevant goals for care and makes sure that all involved parties are aware of personal goals, and that each person’s role relative to goals, is clear. Theoretically, this aligns with quality improvement projects and complexity science where goals/ vision define subsequent plans, process and evaluation [49, 56].
2. The synergetic cyclical care process: In the PACT intervention, the three PCC, IC and Pro-C elements synergistically build on each other. We hypothesise that Pro-C is most influential for health and functional outcomes, and that Pro-C relies on the presence of Goal-PCC and IC to achieve effects. The design of a care plan follows a cyclical pattern in line with quality improvement and complexity theory: The development of goals, care plan and delivery continuously adjust to new insights and feedback from the involved parties. The care process is described in more detail elsewhere (Additional file 1 – the PACT care process). Briefly, the PACT team supports usual care in the creation of a goal-oriented, person-centred, integrated and pro-active care plan according to the following simple rules.
  1. PCC, where “what matters” to the person drives decision making of the care plan.
  2. IC includes comprehensive care planning by a multi-disciplinary, cross-organisational care team, designed to meet goals from 1. The process involves the person, the PACT mini-team (see below) and the informal and formal long-term carers who will take over when PACT withdraws. It results in a negotiated care plan, which sets out the overarching personal goals (what matters), and then outlines roles and responsibilities for the long-term team: who does what, when, and why. PACT is mindful that a strong care plan ownership by both patient and the most involved health workers is essential for long-term success after PACT withdrawal.
  3. Pro-C includes risk management and preparation for early release to as low-level care as possible while ensuring that adequate evidence-based guidelines, such as comprehensive geriatric assessment [63], have been reviewed. If possible and desirable, PACT implements guidelines for the range of conditions present. Included in the team’s repertoire are support for self-management, emergency contingency plans, diagnosis specific monitoring tools (i.e. signs of infection in COPD patients, weight increase in heart failure patients), and the “bow-tie” analysis [47]. To be included in the care plan, pro-active measures must align with personal goals from 1 and the advice of the multi-disciplinary team from 2.
  4. Continuous evaluation of both care planning and delivery, together with the person, to ensure dynamic learning and adjustment of the process in accordance with “what matters”. The patient’s feedback regarding the realisation of “what matters” is the guiding principle.
  5. PACT starts the delivery of care while usual care resources are still being assembled/trained. PACT aspires to a gradual handoff of care delivery to self-management and usual care as soon as possible. PACT revisits the patient and his/ her care team for up to 3 months after withdrawal. If significant gaps in planned care occur during this time, PACT either temporarily fills the gap or re-assesses the plan to set it on course again.

A core team consisting of a nurse coordinator, a physician and a secretary review all referrals. Patients admitted to the intervention are assigned a PACT mini-team consisting of a nurse, physician, physiotherapist, occupational therapist and pharmacist. One of these takes on the role as the primary coordinator of the patient pathway for the duration of PACT involvement. The mini-team have the freedom to “invent” the best possible care plan together with the person in question, as long as they are loyal to the above principles. Their work will consist of exploring patient goals, initiating diagnostic work-ups and other relevant evaluations, addressing the immediate clinical needs of the patient, and starting the coordination work regarding identifying and recruiting the usual care resources that need to be involved in long-term care planning and delivery when PACT withdraws. Handoff is thus a longitudinal collaborative process, where PACT works together with the patient and usual care to support them while a long-term care plan/ delivery comes into place. The average active PACT intervention time was 30 days.

PACT has a daily meeting involving all PACT employees, across the mini-teams, where the team members discuss both challenges at patient and system level. The PACT team project leader reports regularly on system level challenges to the PACT cross-organizational board.

### Data sources and quality

The study extracted following variables for eligible populations in the four intervention and control municipalities from the EHR: Age, sex, start and stop time of outpatient, ambulatory care and inpatient episodes of care, degree of emergency for each episode, ICD-10 diagnoses and Diagnosis Related Group (DRG)-codes registered for each episode [64], DRG-points and date of death. Professionals collect these variables as a matter of routine care for all patients. The local hospital, which supplied our EHR-data, provides 97% or more of all out-patient and in-patient hospital services for the local population [65]. All Norwegian hospitals report these data routinely to the Norwegian Patient Registry (NPR). NPR consistently assesses data quality and corrects errors in the source data in collaboration with the source institution [65]. The EHR is updated with mortality data from the Norwegian Population Registry (PR) on a monthly basis, including all deaths (i.e. deaths at home, hospital or other institutions) in the study population. It takes approximately one week from when the physician issues a death certificate until the PR is updated (personal communication from PR).

### Patients

#### Intervention patients – index episode

The analysis includes all patients who consented to (care consent), and were offered treatment by the PACT team from Oct 2014 and Sept 2015 in M1 and M2 respectively until March 2016, with a 6-month follow-up, ending in Sept 2016. As there is no agreed method of identification of patients with CLNs, PACT applied the following recruitment process. General practitioners, home-nursing services, hospital specialists or hospital nurses were encouraged to refer community-dwelling or hospitalised patients with multi-morbidity, complex long-term care needs and high short-term risk for emergency hospital (re-)admission based on their professional discretion (i.e., a clinical crisis).

- Inclusion criteria: Patients living in M1 or M2, aged > 60 years, referred to a PACT-team to be reviewed for eligibility to receive PACT care and the patient provided an oral consent, recorded in the EHR, to receive PACT care (care consent).
- Exclusion criteria: The PACT team reviewed all referrals, and declined patients who had an adequate care plan, or were in no need of a multi-disciplinary follow-up. PACT declined these referrals within < 24 h. They received no PACT services.

#### Control patients – index episode

All PACT referrals are indicative of a clinical crisis in a person with multi-morbidity. Patients > 60 years, who experienced a clinical crisis as evidenced by an emergency admission (index episode), were eligible as controls. Controls were recruited from the pool of similar patients who had not received PACT care in the PACT intervention municipalities (M1, M2), and from two control municipalities (M3 and M4), where the PACT intervention was unavailable. M3 and M4 had a similar population size and local health care structure to M1 and M2 respectively.

Controls received usual care. Usual care consists of evidence based care for the cause of emergency admission to hospital. Depending on the patient’s condition, care for other diagnoses is either achieved through referrals to the appropriate in-house specialist service, or through a recommendation for follow-up in the discharge letter directed at the GP. Care-coordination and integration at discharge is achieved through standard electronic communication and discharge routines designed in collaboration by the hospital and municipal care organizations.

### Analyses

We followed intention to treat principle, as we included all patients who were offered and gave their consent to receive services from PACT services in analyses, independent of the actual length, content, and delivery of PACT services.

#### Power, modelling decisions and statistical significance

We made apriori power calculations for emergency admissions. These indicated a need for at least 390 patients in each group to show a 20% reduction in emergency admissions, (β of 80% and α of 0,05%). We based power calculations on Norwegian hospital admissions data of patients with at least one admission last year (location parameter μ = 0.5 and scale parameter *σ*=1.1, non-parametric Wilcoxon two-sample test). [66]

To minimise type I error, we based analytic modelling decisions on the size of results rather than statistical significance, reserving statistical tests for evaluation of outcomes. We considered a change in point-estimates > 10% to signify a large enough confounding effect to warrant adjustment for the variable in the statistical model [67, 68]. Similarly, we considered a 10% difference in effects between a main analysis and a sensitivity (sub-group) analysis to be large enough to warrant separate consideration in Results. We defined a probability of < 0,05 as statistically significant. We report both exact *p*-values and 95% confidence intervals to reflect the statistical uncertainty of point estimates. The 95% CI also conveys statistical significance at the chosen significance level, as a 95% confidence interval for a RR which includes 1 means that the null-hypothesis was not rejected. There were no interim analyses planned.

#### Outcome measures

We expected the PACT intervention to have its main effect through its Pro-C component, which prevents the need for emergency care. In the absence of increased mortality, we interpret less use of emergency resources as an indicator of better health and function. We hypothesised that increased use of planned care events at the lowest effective level of care would be instrumental in the reduction of high-level emergency care. Although death is the ultimate health outcome, we did not expect a substantial effect on mortality, as the literature did not support this [16]. We monitored deaths mainly to ensure that the mortality experience in the two groups was comparable and that the expected reductions in care utilisation was not attributable to increased mortality. Based on these expectations, we chose the following outcome measures within the observation period. Outcome measures by hypotheses were:

- H1: We expect a reduced utilisation of high-level emergency care.
  - Count of emergency admissions (defined as care required within 24 h) – reflects the number of emergencies that could not be solved by municipal care.
  - Sum of emergency in-patient bed days – is a composite measure reflecting both number of emergencies and length of stay resulting from each emergency.
  - Count of re-admissions (emergency admissions within 30 days of last discharge) – is a measure of the quality of the discharge arrangements of the previous hospitalisation.
- H2: Increased utilisation of low-level and planned care:
  - Count of planned out-patient visits
  - Count of emergency out-patient visits
- H3: No change in mortality risk:
  - Mortality risk at three and six months follow-up

The follow-up started at the time of the first referral to PACT (intervention) or at the time of emergency admission (controls) and ended at six months follow-up or death. Administrative personnel recorded outcomes as a matter of routine and were blind to group allocation.

#### Matching, propensity score, and balance

This is parallel arm study, with a 1:2 intervention-control ratio. We identified both a local (same municipality) and a distant control from a similar municipality without PACT services, for each intervention patient. In the pooled data, we worked with matched triplets: The intervention patient + their local + distant control.

Within the eligible control populations, we performed exact matching on sex, intervention-control site and year of the episode. Within these constraints, we identified controls with a propensity score of +/− 0,2 SD calliper of the intervention patient’s score using MatchIt software following the methodology laid out by Rubin and Rosenbaum [51, 52, 69, 70]. We used the following variables with a theoretical link to outcomes to build the propensity score: [71, 72] Demographic variables (age, sex), last year’s care-utilisation variables, last two year’s morbidity variables and risk scores reflecting resource utilisation, re-admission risk [73], and mortality.[77] Three per cent of intervention patients lacked hospital visits last year so we could not construct their propensity scores. Within callipers, we matched on Mahalanobis distance (MD) on the three variables that were most unbalanced after PS-matching, as recommended by Rubin [53]. We provide balance statistics in line with recommendations by Rubin [53].

#### Regression analyses

The purpose of regression analyses is to provide an as unbiased estimate of the main effect of treatment variable as possible. Even when there is an excellent balance, there may be residual confounding, which may require additional multivariate adjustment analyses of the matched dataset. In agreement with the data fit, we chose negative binomial regression for our count variables (sum bed days, admissions and outpatient visits) [75] and Cox regression for mortality risk. Using Kaplan Meier (KM) plots and proportional hazards (PH)-test, [76] we found the PH assumptions to be violated at 4–6 months, although the KM-lines did not cross (See Fig. 2). We report RRs for both 0–3 months and 0–6 months. Readers should interpret the latter as an average RR for the full period. We chose robust estimations of standard errors in line with recommendations [75].

Exposure variables: We used both continuous and categorical variables as they were reported in the EHR, with the exception of Lead Days-variable (Number of days in hospital leading up to inclusion). Lead-days had a U-shaped relationship to several outcome variables, which is why we created a quintile nominal variable, and included both the nominal and continuous version in model building. None of the final main effect 95% CI were unreasonably wide, so we did not consider collinearity a problem (See Table 3).

We built models according to a general rule set to minimise the effect of subjective modelling decisions on results. In a combined stepwise forwards / backwards selection process, we examined the confounding effects of variables with a statistically significant difference, or a standardised difference > 0,2 SD between groups and the three compound variables on resource utilisation [64], re-admission risk [73], and mortality [74]. For each outcome, we added the corresponding “before-measure” to all models. For mortality, there are no before measures, so we included proxies for death-risk instead: age and the Elixhauser score [74]. Finally, we added the matched-triplet-ID as a random-effect variable to adjust for dependencies caused by matching [71]. The Higgins I^2^ statistic [77] showed heterogeneity across study sites, which is why we added “Site” both as a random and a fixed effect variable following Cochrane meta-analysis advice [78]. In sensitivity analyses, data from outlier sites were omitted to estimate their effect on results [77].

#### Bias concerns

Controls become eligible upon an emergency hospital admission while intervention patients become eligible upon referral to the PACT intervention. We outline the potential biases due to these differences, and how we have dealt with them in Table 1.

**Table 1:** Overview of bias concerns, consequences and adjustments in the PACT intervention study, Norway 2014–16

Bias type	Description	Exploration/ adjustment of bias consequences
Survival / Lead-time bias	More patients (69%) in the intervention group than in the control group (19%) had Lead Days in the hospital before inclusion. Mortality: Intervention patients must survive lead days to be referred and included in PACT. Survivors may be healthier and cause a survival bias. Sum emergency inpatient bed-days: In controls, we count “inpatient days” from the first day of emergency admission. In PACT patients, we start counting from the time of referral to PACT, leaving out emergency Lead days before referral. If left unadjusted, this would bias comparisons towards a lower sum of emergency days in intervention patients.	Mortality risk analyses: Restricted sub-group analyses to control-patients with a survival time equal to or greater than median Lead-days in the intervention group. Sum emergency inpatient days: We adjusted for Lead Days so that effect estimates are independent of prior lead days. We tried matching on lead-days, which would be the preferred avenue, but we could not find enough matching controls for this. We restricted analyses of sum emergency bed-days to PACT patients with an index emergency hospitalisation so that both groups add emergency bed-days from their index episode to the 6-month outcome measure.
Indication bias	Referral to the PACT intervention is less likely for terminal patients, or patients they judge to be unsuitable for the intervention for other reasons. In the control group, providers are likely to refer all other patients, including terminal patients to emergency admissions who then become eligible to be controls. We have no data, on the judgements made by referring professionals in either group.	Adjustment for possible under-referral of terminal patients to the intervention: We used the Elixhauser death risk score and the modified (m)-PARR30 score for both matching and adjustment. The C-statistic was 0,74 and 0,71 for death within six months in a local hospital population for these two predictors respectively. We made sub-group analyses restricted to control-patients who survived median Lead-days to exclude terminal controls who died in their first few days in the hospital. We estimated crude mortality risk in intervention patients that the PACT-team excluded since these might include terminal patients.
Healthy selection bias:	69% of the intervention and 100% of the control patient index episodes were linked to an emergency admission. Intervention patients who had no index-episode emergency hospitalisation may be healthier than controls.	Sub-group analyses restricted to intervention patients whose index episode was an emergency admission.

#### Presentation of results and software

To explore the bias concerns outlined above, we made the following sub-group analyses for all outcomes: 1) Survival and indication bias: Only controls who survived median lead-days of PACT patients, 2) Healthy selection bias: Only PACT patients who had an emergency admission index episode, and 3) strictest subgroup: a combination of 1 and 2. In the abstract and results, we focus on multivariate-adjusted rate ratios (RR = Intervention Rate/ Control Rate) from the strictest sub-group analysis, as these represent our most unbiased effect estimates. We present results for other groups in Table 3.

The SNOW system© [79] extracted data from the EHR for us. We used MatchIT v 2.4–21© [70] for PS-matching. We used Stata© v 14.0 and 15.0 for statistical analyses. We calculated heterogeneity I^2^ with "Comprehensive Meta-Analysis Version 3.0© [80].

## Results

### Exclusions, balance between groups and study population

**Fig. 1:**
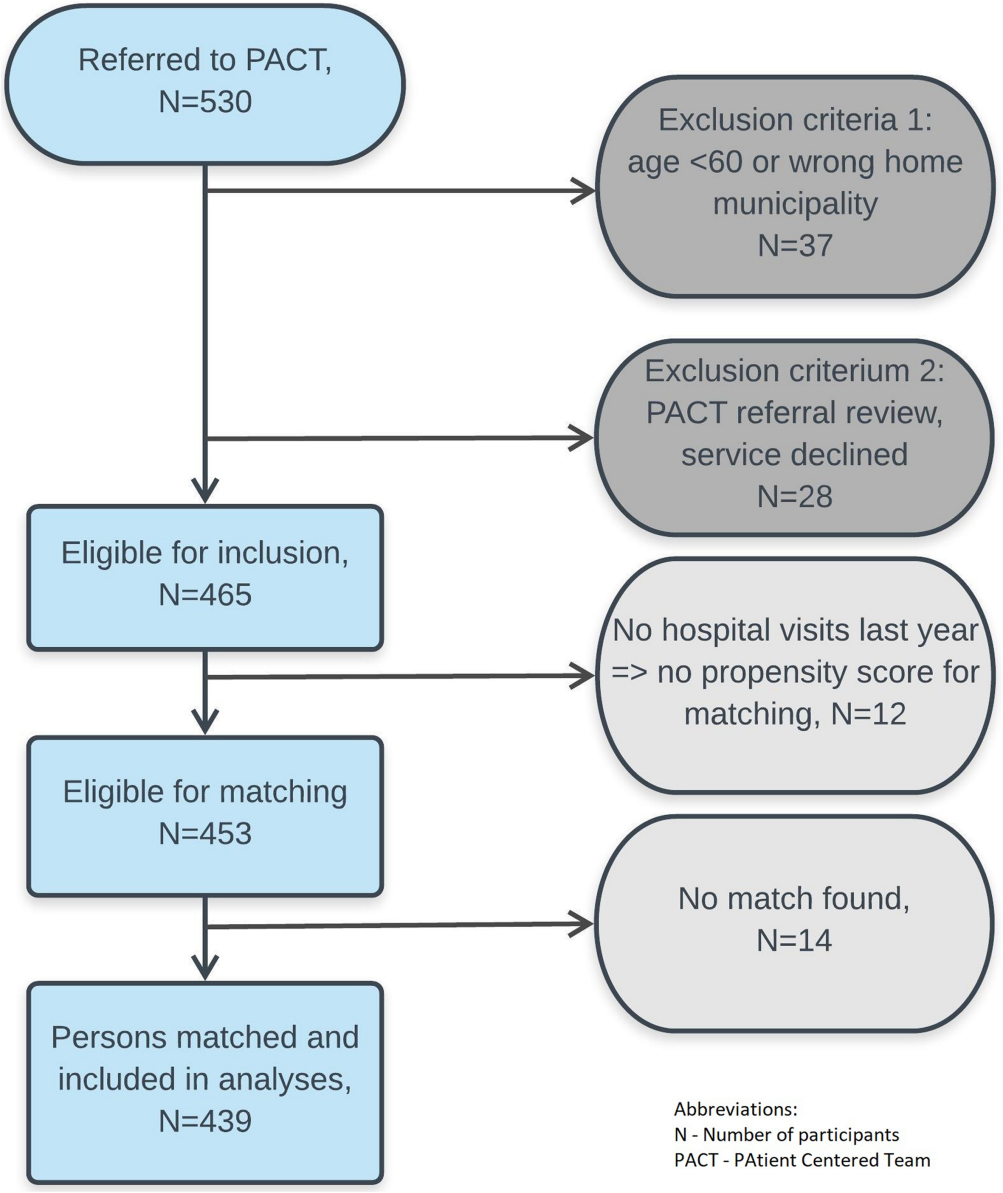
Inclusion and exclusion Flowchart. Legend: The figure shows eligible PACT patients and exclusions at the person level. The Patient-Centred Team (PACT)-study, Norway 2014–16

530 persons were referred to the PACT-intervention for 606 care episodes from Oct. 2014 – Sept. 2016. The PACT-referral review team declined 5 % of referrals. Figure 1 shows the exclusion flowchart. 83% of all referred and 94% of all eligible persons contributed to final analyses.

The dataset showed an excellent balance, with no balance tests outside of the recommended range. Some of the matching variables differed significantly between groups (see Table 2). As expected, the distribution of Lead days, which we were unable to match for, differs between groups.

**Table 2:** Background variables and balance. Crude descriptive measures at baseline, for participants and their matched controls. If not otherwise marked, the point estimate is median and dispersion 5–95%-tile. All variables in the table were matching variables, except for the two Lead Days variables. The Patient-Centred Team (PACT)-study, Norway 2014–16

		Controls			Intervention			p
	Unit	N	Point estimate	Dispersion	N	Point estimate	Dispersion	
Sex (%)	male	779	41%	NA	439	41%	NA	0.51
Year at inclusion (%)	in 2015	779	51%	NA	439	51%	NA	0.98
Age (mean/SD)	years	779	78.81	8.68	439	80.02	8.72	0.02
m-PARR30. 2Y (mean/SD)	Score	779	2.19	0.57	439	2.16	0.61	0.49
DRG points. 1Y	Sum	779	2.20	0.03–12.65	439	2.70	0.32–14.79	0.10
# Main diagnoses, 1Y	Count	779	3	1–8	439	3	0–8	0.30
# Bi-diagnoses 1Y	Count	779	3	0–13	439	3	0–12	0.05
# Long-term Diagnoses.	Count	779	11	2–29	439	11	3–28	0.41
m-PARR30, 2Y	Score	779	2.15	1.30–3.16	439	2.09	1.33–3.20	0.10
Elixhauser, 2Y	Score	779	5	0–20	439	5	0–20	0.28
Emergency Inpt Adm. 1Y	Count	779	2	0–8	439	1	0–7	0.05
Emergency Inpt Adm, 30d	Count	779	1	0–4	439	1	0–2	0.96
Emergency Bed days, 30d	Sum	779	2	0–15	439	3	0–16	0.03
Emergency Bed days, 1Y	Sum	779	6	0–52	439	6	0–55	0.86
Emergency Outpt visit, 30d	Count	779	0	0–1	439	0	0–1	0.43
Emergency Outpt visit, 1Y	Count	779	0	0–3	439	0	0–3	0.95
30d Readmissions, 1Y	Count	779	0	0–1	439	0	0–2	0.26
Planned Inpt Adm, 1Y	Count	779	0	0–4	439	0	0–2	0.04
Planned Inpt Adm 30d	Count	779	0	0–1	439	0	0–1	0.74
Planned Outpt visit, 1Y	Count	779	2	0–21	439	2	0–18	0.22
Planned Outpt visit, 30D	Count	779	0	0–3	439	0	0–3	0.06
Lead Days	Count	779	0	0–9	439	4	0–30	< 0.001
Quintile Lead Days (%)	Q 1 + 2	779	78%	NA	439	20%	NA	< 0.001

*Abbreviations*: *N* Number of patients, *#* Number of, p-probability, *SD* Standard deviation, *m-PARR30* modified PARR score [76], Elixhauser score – Elixhauser Comorbidity Measure [77], *DRG* Diagnosis Related Groups [64], Readmissions – New emergency admission within 30 days of last hospital discharge, *Inpt* Inpatient, *Adm* Admission, *Outpt* Outpatient. Main-diagnosis: The current diagnosis which caused admission. Bi-diagnoses: Other diagnoses relevant for the care of the current problem

Time spans: 1Y - Last year prior, 2Y – Last 2 years prior, 30d – Last 30 days prior

Statistics: *P*-values for two-sided t-tests (continuous normal variables) Wilcoxon rank-sum test (continuous non-normal variables) and Chi2-test (discrete variables) for difference between control and intervention groups

### Hypothesis 1: reduction in emergency care utilisation

All adjusted RR indicated a reduction of high-level emergency care utilisation.

In our strictest sub-group analysis, RR for emergency admissions was 0.90 (95%CI: 0.82–0.99), RR for sum emergency bed days was 0.68 (95%CI: 0.52–0.79) and RR for 30-days readmissions 0.72 (95%CI: 0.41–1.24). See Table 3 for details of multivariate analyses in other groups.

**Table 3:** Rate Ratios for outcomes: Multivariate multilevel adjusted pooled analysis, at six months follow-up, by outcomes and sub-group. Negative Binomial regression for health care utilisation outcomes and Cox regression for mortality. The Patient-Centred Team (PACT)-study, Norway 2014–16

Outcome	Population sub-groups	N	RR	p	95% CI	
					Lower	Upper
Count, Emergency Admissions after index episode	All, adjusted	1218	0.95	< 0.001	0.94	0.96
	Only controls surviving Lead days (1)	1195	0.95	0.024	0.91	0.99
	Only PACT patients with index emergency hospitalizations (2)	856	0.90	0.018	0.82	0.98
	Combination of 1 and 2	838	0.90	0.033	0.82	0.99
Sum, Emergency Bed days (including index episode bed days)	Only PACT patients with index emergency hospitalizations (2)	856	0.62	< 0.001	0.49	0.77
	Combination of 1 and 2	838	0.68	0.005	0.52	0.89
Count, emergency readmissions within 30 days of discharge, after index episode	All, adjusted	1218	0.64	< 0.001	0.52	0.78
	Only controls surviving Lead days(2)	1195	0.63	< 0.001	0.51	0.79
	Only PACT patients with index emergency hospitalizations (2)	856	0.71	0.213	0.41	1.22
	Combination of 1 and 2	838	0.72	0.231	0.41	1.24
Count, Planned outpatient visits	All, adjusted	1218	2.40	< 0.001	2.21	2.61
	Only controls surviving Lead days(1)	1195	2.41	< 0.001	2.22	2.62
	Only PACT patients with index emergency hospitalizations (2)	856	2.26	< 0.001	2.01	2.54
	Combination of 1 and 2	838	2.27	< 0.001	2.02	2.55
Count, Emergency Outpatient visits	All, adjusted	1218	0.82	0.001	0.73	0.92
	Only controls surviving Lead days(1)	1195	0.82	< 0.001	0.76	0.88
	Only PACT patients with index emergency hospitalizations (2)	856	0.89	0.408	0.67	1.18
	Combination of 1 and 2	838	0.90	0.464	0.68	1.20
Mortality 0–3 months	All, adjusted	1218	0.38	< 0.001	0.24	0.60
	Only controls surviving Lead days(1)	1195	0.46	0.001	0.28	0.73
	Only PACT patients with index emergency hospitalizations (2)	856	0.32	< 0.001	0.19	0.55
	Combination of 1 and 2	838	0.39	0.001	0.22	0.70
Mortality 0–6 months	All, adjusted	1218	0.53	0.001	0.37	0.77
	Only controls surviving Lead days(1)	1195	0.60	0.010	0.41	0.89
	Only PACT patients with index emergency hospitalizations (2)	856	0.48	0.003	0.30	0.78
	Combination of 1 and 2	838	0.57	0.028	0.34	0.94

*Abbreviations*: *N* Number of patients, *RR* Rate Ratio (Rate Interv /Rate Control), *p* probability, *95% CI* 95% confidence interval

Sub-group analyses: (1) Only controls surviving Lead days: Controls who survived the intervention group’s median lead days in the hospital. Excluded: Controls who died during the first 4-5 days and their matches. (2) Only intervention patients with an index emergency hospital episode. Excluded: Intervention patients with index episode in the municipality or planned hospitalisation and their matches

Final model adjustment variables: Emergency admissions: Fixed effect: Count of emergency admissions last year, Site. Random effect: site, triplet-stratum ID.

Final model adjustment variables: Sum emergency inpatient days: Fixed effect: Quintile of lead days, Sum emergency bed days last year, Site. Random effect: site, triplet-stratum ID

Final model adjustment variables: Count 30-day Readmissions: Fixed effect: Quintile of lead days, Count re-admissions last year, Site. Random effect: site, triplet-stratum ID.

Final model adjustment variables: Planned outpatient visits: Fixed effect: Count planned outpatient visits last year, Site. Random effect: site, triplet-stratum ID

Final model adjustment variables: Emergency outpatient visits: Fixed effect: Quintile lead days, Count emergency outpatient visits last year, Site. Random effect: site, triplet-stratum ID

Final model adjustment variables: Mortality 0-3 months: Fixed effects: Quintile lead days, Age, Elixhauser score, Site. Random effect: site, triplet-stratum ID

Final model adjustment variables: Mortality 0-6 months: Fixed effects: Quintile lead days, Count readmission last year, Age, Elixhauser score, Site. Random effect: site, triplet-stratum ID

### Hypothesis 2: increased utilisation of low-level planned care

The adjusted RR indicated a substantial increase in planned low-level care and no change in low-level emergency care.

The RR for planned outpatient visits was 2.3 (95%CI: 2.02–2.55), while RR for emergency outpatient visits was 0.9 (95%CI: 0.68–1.20) in our strictest sub-group analysis. Please see Table 3 for details of multivariate analyses in other groups.

### Hypothesis 3: unchanged mortality rates

The adjusted RR indicated a reduction in mortality risk at both three and six months follow-up.

In all, 74 (17%) and 180 (23%) patients died within the 6-month follow-up in the intervention and control group respectively. The median survival time in both the control and intervention groups was 182.5 days (5–95%-tile Controls: 12–182.5, PACT: 35–182.5, *p* = 0.009).

**Fig. 2:**
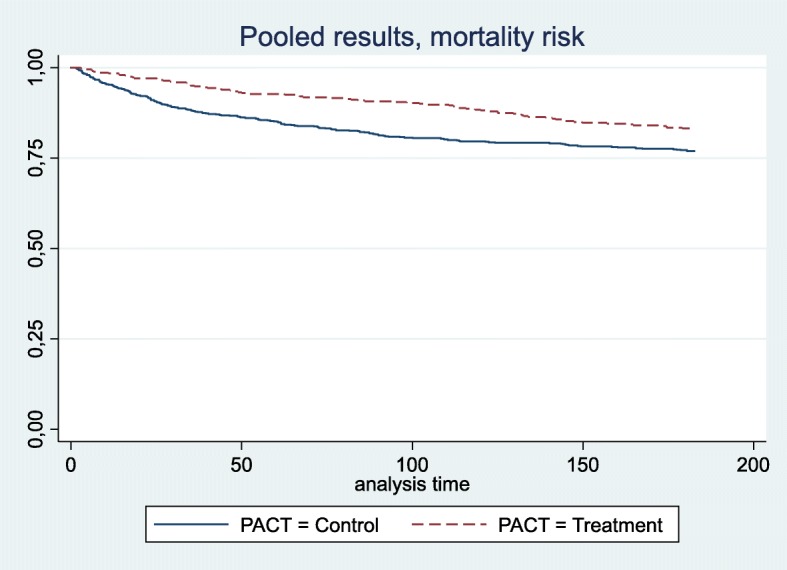
Mortality. Legend: Crude Kaplan-Meier curves, showing the proportion of patients alive by time, and group in 6 months follow-up period. Pooled data, (*N* = 1218). The Patient-Centred Team (PACT)-study, Norway 2014–16

The RR for death at three months was 0.39 (95% CI: 0.22–0.70) in our strictest sub-group analysis. The protection waned somewhat after three months so that at six months the RR was 0.57 (95% CI: 0.34–0.94), also in our strictest sub-group analysis. Please see Table 3 for details of multivariate analyses for other groups. The Kaplan–Meier plot shows a net survival benefit in the intervention groups by the end of the follow-up period (Fig. 2).

There were in all 28 referred patients who did not get the PACT intervention within 24 h and were therefore ineligible for analyses. Of these 6 (21%) died. There were in all 4 (15%) deaths among the 26 persons who did receive the intervention but were excluded from analyses due to technical reasons (no prior hospital history, no suitable match). Small numbers preclude drawing any conclusion from these numbers.

### Sub-group and sensitivity analyses

#### Survival and indication bias

When we compared RRs of “All, adjusted” with that of “Only controls surviving Lead days” we saw a weakening of the three month RR mortality from 0,38 to 0,46 (Δ16%) and six month RR from 0,53 to 0,66 (Δ11%) (Table 3). The result implies survival and indication bias, but the intervention effect was still substantial. A Kaplan Meier plot of the strictest sub-group, excluding outlier sites also showed a net benefit for the intervention group. (See Additional file 2) No other outcome changed > 10%.

#### Healthy selection bias

We saw a strengthening of the three month RR estimate from 0,38 to 0,32 (Δ19%) when comparing RRs for “All” with “Only index emergency hospitalisations” (Table 3). The finding weakens the case for healthy selection bias. No other outcome changed > 10%.

#### Lead time bias

The crude RR estimate of sum-emergency days changed direction from a crude RR of 1.1 to an adjusted RR of 0.58 upon adjustment for Lead days, showing Lead days to be a strong confounder for this outcome (data not shown).

#### Heterogeneity and interactions with site

There was no evidence of interaction across intervention sites, as the interaction term between intervention site and outcome were not significant for any outcomes (data not shown). Except for 30-day readmissions, all outcomes displayed significant heterogeneity across control sites, but the control sites took turns in being the outlier for each outcome (See Additional file 2). Exclusion of outlier sites changed the 6 month mortality RR point estimates for one sub-group analysis: “Only index emergency hospitalisations” from a RR of 0,48 (*p* = 0,003) to a RR of 0,54 (p = 0,01) (Δ11%). No other outcomes changed > 10%.

## Discussion

### Principal findings

Compared with propensity score matched controls, the care process of frail multi-morbid elderly who received the PACT intervention had a reduced risk of high-level emergency care, increased use of low-level planned care, and substantially reduced mortality risk.

### Strengths and weaknesses

#### Internal validity

As administrative personnel was unaware of the treatment status, systematic misclassification of outcomes is unlikely. The high degree of completeness of data makes missing outcomes a negligible problem [65], and strengthens internal validity.

Bias evaluation: We have discussed some possible biases that might have influenced our analysis. Admittedly, analyses restricted to controls who had survived the median lead-time showed a weaker, albeit still significant mortality risk reduction. It is relevant to point out that this sub-group analysis is, an “over-correction” because by definition half of the intervention patients will have survived a shorter lead-time than the median, whereas all the controls survived the lead-time period. The strictest sub-group analysis, combining adjustment for both indication and healthy selection bias, did not erase our findings.

We are naturally unable to account for unknown biases or confounding, as they would have been in an RCT. We have no systematic review of referral practices. The care process documentation is limited to changes in the health care utilisation outcomes, which did support our hypotheses, although a more detailed process evaluation is called for. Both challenges are limitations, and areas for future research (see below).

#### External validity

Non-parametric pre-processing by use of propensity score matching allows a post hoc creation of a control group and reduces model dependencies [81]. Although considered to have a lower internal validity than an RCT, its applicability to “real world” data gives a higher external validity [82]. The balance measures were excellent, warranting comparability between groups on all known variables. The matching software program (MatchIt) had no access to follow-up data, which ensured that matching was not influenced by outcomes data, which is a critique towards this methodology [83].

Since we were granted permission to extract routine data without formal consent, we were able to include both patients with cognitive impairment and/ or acute morbidity. Both conditions contribute to the underrepresentation of elderly in clinical research [84]. Exclusions of these highly vulnerable patients from outcomes evaluations, represent an ethical challenge, as these populations have an equal right to quality assurance and evaluation of care as other patient groups [84].

Norwegian health care constitutes its own context, which probably differs from that of other countries in important respects. We have one study that shows low integration of care in Norway [40]. The context dependency is an area worthy of further research (see below). Within Norway, we implemented the intervention at two sites and found no interactions by Site. Effects did vary by control-context, but the intervention effect was significant even after we had adjusted for control-site variation as a random effect. The study has high external validity, which is a strength.

### PACT results in light of literature

#### PACT compared to other goal-oriented PCC interventions

The Goal-oriented PCC care of PACT has still no internationally recognised vocabulary. However, similar approaches are “flipped care” [30], and “personalised care planning” [15]. Their commonality is the construction of personalised goals together with the patient, which subsequently drives care planning, care delivery, and care evaluation. “What matters” is the organising principle of care, inescapably present throughout the entire care process. Our PCC approach differs fundamentally from other person-centred approaches linked to integrated care, where authors often describe tailoring of care plans to personal values, needs, and preferences, yet they fail to show how plans, care delivery and evaluation become shaped by the person’s goals [15].

We have found only one controlled clinical trial directed at persons with CLNs which applies “flipped healthcare” similarly to us. Sweeney compared a “Patient-Centred management” with usual case-management for cancer patients at the end of life and found reduced care utilisation and costs, with no shortening of life [85]. Coulter’s Cochrane review on personalised care planning reports a small but significant improvement in bio-psycho-medical health, which “… *appear greater when the intervention is more comprehensive, more intensive, and better integrated into routine care*.” However, she found no eligible studies on multi-morbidity in the review, which makes her promising findings tangential to our work. Two protocols that subscribe to a goal-oriented PCC care process show that Goal-PCC is an up and coming concept [86, 87].

#### PACT compared to interventions aligned with the chronic care model

We also compared PACT with comprehensive care interventions directed at persons with CLNs, defined as at least two components of the Chronic Care Model (CCM) [16]. Of 15 studies that reported mortality risk, 3 reduced mortality significantly [88–90]. Two of the three studies are from Norway [89, 90]. In a comparative study across 11 nations on gaps in care integration, Norway performed poorly [40]. As there is a lack of follow-up studies, at this stage we can only speculate on whether this is why the Norwegian context seems to be more responsive to comprehensive care interventions. However, it is difficult to see any clear pattern of how the three positive studies differ from the 12 negative studies. de Bruin concluded in her review that *“No evidence was found for a beneficial effect of comprehensive care on ( …*) *mortality.”* de Bruin also notes the diversity in effects from comprehensive care programs, and links it to the variation in interventions and context [16].

We found two other studies that resembles the PACT study, in that they apply a three-component PCC, integrated and pro-active care model. Yet neither of these had paid close attention to the synergies between the three elements. Neither study showed any effects on hospital health care utilization, costs or function. This is in line with several recent reviews of multi-component comprehensive care models designed to address the challenges of multi-morbidity or frail patients, where the results across studies are mixed [21, 22, 91–93]. The reviews lament the heterogeneity of both target groups and intervention designs. We believe a careful untangling of the desired goals, and the chain of causes and effects that lead to the desired goal at both population and individual levels are not yet well enough understood.

#### PACT mechanisms for effect

The PACT intervention did not introduce any new biological treatments. The mechanisms for a mortality difference between the groups must either be the result of bias between the groups, the result of the intervention or a combination of both. While we have discussed possible biases at great length above, we here present some of the mechanisms of the intervention, which we hypothesise, might contribute to our results.

- **Patient detected quality challenges**: The continuous dialogue with the patient on “what matters to you” give insights into challenges that only the patient can detect, and allows the team to address them effectively. Examples are gaps in care, where delivery of planned care failed, such as missing referrals or missing prescriptions, adverse events that could influence patient willingness to follow self-management advice, i.e. a hypoglycemic episode following recommended exercise, or ineffective pain management, which hinders mobility training.
- **Proactive care planning**: Comprehensive geriatric assessment includes an early evaluation and management of common geriatric complaints, such as exercises in sarcopenia, and effective under-nourishment management [63]. We added several complementary risk-management strategies, (see intervention design) designed to stabilise the health situation and prevent further clinical crises.
- **Avoid iatrogenic disability**: frail elderly are vulnerable, at risk for a large fall in function following minor insults [63]. Hospitalization of elderly patients is associated with delirium, decreased mobility and increased dependency [94]. PACT works to mitigate decompensation through early discharge to a familiar environment.
- **Prescription review:** performed by the pharmacist revealed errors and interactions in a majority of the medication lists reviewed. The pharmacist corrected such errors in collaboration with the relevant professionals.
- **Improved care delivery according to plan**: Due to fragmented information and organisational systems, professionals have few tools to support the interdisciplinary dialogue and collaboration. Typically, no professional will have the full overview of the needs or activities for patients with CLNs. Gaps and overlaps are virtually “invisible” in a silo-based system. The PACT team has the time and capacity to make an overview of all care, facilitate dialogue across organisations and professions and continuously update all parties involved in the care plan. PACT ensures that the care plan aligns with the patient’s personal goals, planned activities are not at odds with one another, and care plans translate into actual care delivery.
- **Professional motivation**: The goal-oriented PCC serves to make all parties work towards the same goal. It not only makes sense to the person who needs care. Working together with the patient towards “What matters” seems to have a substantial motivational effect on involved professionals across organisations in the primary and second care sector [32]. PACT team members feel they are “making a difference”, which may in itself influence outcomes.
- **Flexible and adaptive goal driven care**: Finally, PACT does not force care planning into a specific method or format. Instead, the agents follow a set of principles and pragmatically apply them to reach the negotiated goals. We hypothesise that this flexibility allows the partners to create tailored solutions, which may be more effective than multiple un-coordinated standardised pathways for multi-morbid patients [26].

PACT builds on real-world data, has shown transferability across two intervention sites, and produce consistent results across control sites. The PACT project aimed to remedy universally acknowledged challenges [1–4]. Our health care utilisation measures show that PACT succeeds in reducing the emergency care and increase planned low-level planned care. Our underlying hypotheses for why our intervention works are in accord with the literature [9–14]. To summarise, the substantial effects of the “real world” PACT study, concerning both mortality and health service utilisation is unusual.

### Implications for practice and further research

Several comparable interventions in the literature pro-actively identify frail patients in GP EHR, but these studies fail to show effects [95, 96]. It may be, that it is not enough to capture frailty, as some very frail persons may be balancing their lives well enough, and interventions may upset their precarious balance. Finding formal methods of capturing the frail person as they move to an unstable state, while there is still time to regain balance, is an important research question. A formal definition of the PACT target population would also allow us to evaluate how outcomes for a formally identified frail elderly in intervention municipalities compared to the same group in control municipalities.

To further examine external validity in an international context, a comparison of the Norwegian results with international propensity score matched control-groups is an aim for future research.

Although we attribute effects to our theoretical underpinnings, the study merits further evaluation of the causes of death in the two treatment groups, to understand better how effects are produced [97]. A stronger understanding of the care process and mechanisms of effect and how patient pathways differ between groups and contexts would help us understand which components require high fidelity in any intervention site, and which components can and should tailor to the local context.

## Conclusions

The PACT intervention lowers the need for high-resource emergency care, increases use of planned low-resource care and protects vulnerable patients from death compared to propensity score matched controls. We have used a robust methodology and have adequate statistical power to support our findings. The PACT study has designed a person-centred, integrated and pro-active care model, which attends to, responds to and is loyal to “what matters to you?”, using the cyclical goal-oriented adaptive solving strategies from complexity theory. We argue that our study is a contribution towards a better understanding of care for persons with CLNs. Further studies of the effects of goal-oriented PCC may force the highly needed paradigm shift that will make genuine goal driven PCC the norm.

## Additional files

Additional file 1: The PACT care model. A stronger and more detailed qualitative description of the PACT intervention. (DOCX 210 kb)

Additional file 2: Supplementary analyses. This file includes additional tables showing: Crude results for all outcomes. Pooled adjusted results for all outcomes, showing I2 (the heterogeneity index across sites), with outlier-sites first included and then excluded. The Kaplan-Meier plot for mortality, with outlier sites excluded. (DOCX 53 kb)

## Data Availability

Due to strict Norwegian privacy, data cannot be shared openly. Data can be made available for external researchers if approval from the Norwegian Regional Ethics Committee (REC) is obtained. Please contact the corresponding author for assistance to apply for an approval from REC. REC can be also contacted directly regarding the project´s approvals (#2014/1707/REK Sør-Øst A).

## Abbreviations

CLN: Long-term and Complex Needs
DRG: Diagnosis Related Group
EHR: Electronic Health Record
Goal-PCC: Goal oriented Person-Centred Care
IC: Integrated Care
KM: Kaplan Meier plot
Lead Days: # of days in hospital leading up to inclusion in the study
M1 and M2: Municipality 1 and Municipality 2
MD: Mahalanobis Distance
m-PARR30: modified PARR30 score, UK specific coefficients omitted
NPR: Norwegian Patient Registry
PACT: The PAtient Centred Team
PCC: Person-Centred Care
PH: Proportional Hazards
Pro-C: Proactive Care
PS: Propensity Score
REC: Regional Ethics Committee
RR: Rate Ratio
