# Supplementary material for: Person-centred, integrated and pro-active care for multi-morbid elderly with advanced care needs: a propensity score-matched controlled trial
Source: BMC Health Serv Res. 2019 Oct 3;19:682. doi: 10.1186/s12913-019-4397-2 (PMC6777026; doi:10.1186/s12913-019-4397-2)

# Description of The PAatient-Centred Team (PACT) models and methods

By project managers Monika Dalbakk and Lisbeth Spansvoll.

## Purpose

The purpose of this description is to clarify what PACT is, what different PACT models we are testing and what methods the PACT teams use to contribute to a person-centred, integrated and proactive health service for each individual patient, beyond today's healthcare systems. This description should be seen in context with the PACT flowchart (see attachment).

## Background

The PACT project was established in the autumn of 2013 with the aim of strengthening the healthcare service for patients with complex and chronic diseases. The project has drawn upon a patient driven evaluation of care {Berntsen, 2014 #9090}. Through a joint interdisciplinary team across hospitals and municipalities, the teams should contribute to early assessment and diagnosis of patients in their homes and early supported discharge and follow-up of patients recently discharged from the hospital. Patients and relatives are actively involved in planning throughout their care pathway.

# Models and methods

## Organizational models of PACT

During the project period, three variations of the PACT model are being tested:

1. PACT Urban municipalities ( M1 and M2 data included in the study) are larger municipalities with a local hospital: The interdisciplinary teams in M1 and M2 are organized across hospital and municipality, i.e., the teams are established as a joint unit, with employees from both hospital and municipality. The teams are located at the local hospital, but their service is ambulatory. The teams meet the patient both at home, at municipal institutions and in hospitals.

2. PACT Rural municipalities (data from these are not included in the study because they started in 2017): Two municipalities who both have < 6000 inhabitants and are located approximately 1,5 hours drive from their local hospital. The rural municipalities appointed contact persons (professionals, patient coordinators and local teams) who cooperate locally in each municipality with the established PACT team in M1. The interaction is mainly through telephone and video conferencing. The PACT team in M1 meets the patients while in hospital, while the local professionals/teams follow up the patient in the patient's home.

3. PACT Intermediate sized municipality: (data from this municipality is not included in the study because they started in 2018. This municipality has approximately 12 000 inhabitants and is located 2-3 hours away from the local hospital. The local hospital and the municipality have established a cross-organisational team, at the local District medicine centre (DMC). This team is ambulatory and meets the patient in the patient's home, municipal institutions and at the local DMS. The local team collaborates with the hospital staff members of the PACT team in M1 when the patient is admitted to UNN when there is a need of interdisciplinary follow-up from the hospital or a need for internal coordination of hospital services.

## Methods

The methodology developed in the project is based on three main components that align with the Chronic Care Model (CCM); the healthcare services must be person-centred, integrated and proactive


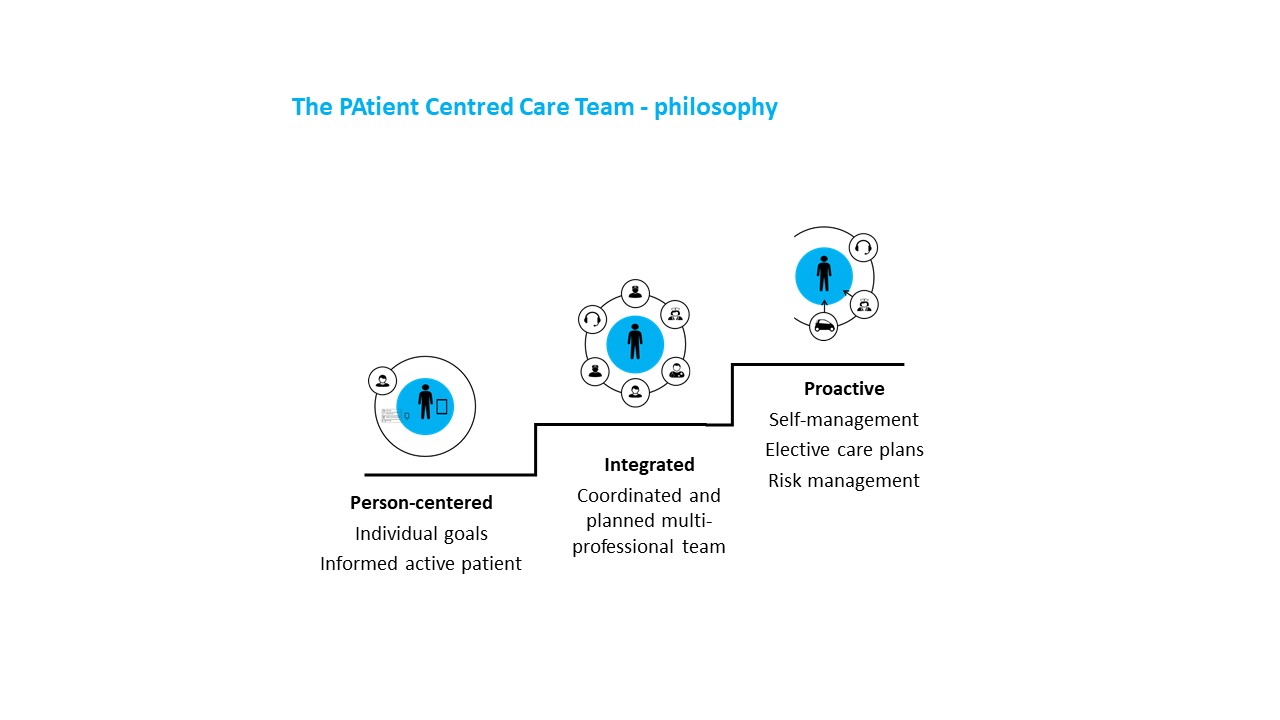


Throughout the project period, the PACT project has outlined these three key elements, and the established teams have developed the working methods their teams use today. It must be clear that PACT is a small part of the overall healthcare service. A complete patient-centred healthcare service can only be achieved when the healthcare service is fully adapted to the Chronic Care Model. For this, changes and development in several parts are required, which is beyond PACT's mandate. The working method developed by PACT must be considered as a methodology, based on how the healthcare service is organised and is functioning today.

Each patient pathway where PACT is involved will be individually adapted, and the processes will most likely vary significantly for each. Despite this, an attempt has been made to visualise a step-by-step description of PACT working methods. This description below should be seen in context with the PACT flowchart (see attachment).

The professionals in the PACT team have participated regularly in workshops where the principles above are discussed, clarified and improved. The toolbox available to the team is in constant development, while the principles stay stable. This allows modification and tailoring not only to the unique patient, but also to the unique circumstance of each intervention site, the resources available to them and their historical and cultural context.

## PACT methods

### 1. Referral

PACT depends on other healthcare services to assess the need for involvement of PACT. Patients are referred, based on consent, to PACT by electronic referrals from GP’s, internal referrals from hospital wards or telephone inquiries and written referrals in Profil (the municipality electronic health record), and sent by home care nurses. Patients and relatives can also contact the team by a telephone inquiry. In those cases, PACT asks the patient for consent to contact the GP, homecare service and other relevant agencies as needed.

### 2. Interdisciplinary assessment of referral

The referrals to PACT are considered and handled by the interdisciplinary team itself. The PACT physician, as the medical professional responsible, handles the references in DIPS (the hospital electronic journal). Physician, nurse, patient coordinator and secretary participate in the first interdisciplinary assessment meeting. Physiotherapist, occupational therapist and pharmacist are included when needed. A note is documented in the hospital and municipality EHRs, regarding PACT's acceptance of the referral to the team.

### 3. Allocation of a mini team

If the referral is considered relevant regarding services from PACT, the patient is assigned a PACT mini-team consisting of a nurse, physician, physiotherapist, occupational therapist and pharmacist. The involvement of the individual professionals depends on the cause of the referral and findings in the initial mapping. One of the professionals in the mini-team gets the role of the patient's contact person and coordinator throughout the patient pathway, for the duration of PACT involvement.

### 4. Obtaining information and patient history

As a preparation ahead of meeting the patient, the mini team reviews background information and status, according to professionals, from the available electronic journals. Since the team have access to both primary and secondary care records, the team can rapidly assess the available information. Further information, also from other agencies, is obtained along the way as needed.

### 5. Meeting the patient

It is essential for PACT to meet the patient as early as possible, preferably a face-to-face meeting, but meetings also are facilitated by video conferencing (between professionals in distant municipalities and the patient when admitted to UNN or at home). At the first meeting a conversation, mutual dialogue and a systematic survey of the patient's goals are started. PACT experiences that finding out what matters most to the patient is a process, that often takes some time and requires several conversations. A first start is to ask the patient to, with his own words, describe what has happened, and how the patient sees his/ her future. PACT has elected not to use a questionnaire or a specific method to elicit “what matters” to the patient, although some of our team members use Motivational interview techniques when appropriate. We find that the most important is to improvise in a sensitive and empathic manner, starting with whatever is brought to the foreground by the patient. In the first interview, the patient's contact person participates from the PCT mini-team and, usually another team member. If necessary, trust and confidence are gained through repeated conversations between the patient and the PACT-contact to support the process of clarification regarding what matters. Both in bio-medical but also psychosocial developments will influence this process. Other participants from PACT meet the patient later in the process. Other actors in the healthcare service are contacted as needed.

### 6. Method for development of patient goals:

We start by understanding “ What is important to you?”

Keywords:

• What has happened and what are your experiences of this situation so far?

• What do you consider the most significant challenges in the future?

• What opportunities do you see ahead?

• What are your objectives and wishes for the future? Is it possible to divide broad, long-term goals to smaller, progressively achievable intermediate objectives?

• What needs to happen, for you to achieve your goals?

PACT has learned, that the more recent the acute incident (for example emergency hospitalisation), the more guidance and involvement from the team is needed for clarification of “what matters” and the development of personalised goals. PACT seeks information from the other involved healthcare professionals, if there are plans that are available, and whether these are still relevant. The existing plans are often specific to a discipline or a single problem. These plans are discussed with the patient, and different options and possibilities of how to move forward with a unified plan are discussed. It is essential that patient and relatives be actively involved, even though they often express a wish for PACT to take control of the management of the process because the healthcare organisation is often overwhelming and unclear. Any dependents of the patient are involved if that patient wishes it to be so.

What matters to the patient, and the patient's goals and objectives derived from “what matters” are documented in both the hospital and the primary care EHR. Today, useful tools that could make it possible to highlight the patient's goals clearly in all a shared medical record is still not available but will be an essential support for a future person-centred care system.

### 7. Interdisciplinary mapping

The professionals in the mini-team meet to discuss the different findings of their assessment of patient goals, needs, values and preferences. The PACT-contact person stays in contact with the patient, while the whole group of involved professionals meet each other during the process whenever needed. The mini-team summarises findings, assessments and draw interdisciplinary conclusions, based on the patient's goals and objectives.

### 8. Involvement of other actors

Once the patient’s goals and targets have been clarified, the PACT mini-team collects current information and assessments from other parts of the healthcare service and invites to dialogue, based on the patient's objectives. Assessments and experiences from the current hospital ward, general practitioner, home care service, and municipal decision authorities are gathered. PACT strives to involve relevant actors to agree on a joint comprehensive plan, hereafter referred to as the treatment and follow-up plan.

PACT has realised that it is not appropriate that PACT prepare the overall plan on their own (based on obtained information), to share it with the other actors. It is essential that those who are going to follow up the plan have ownership of the plan and that it is designed with the same system and methodology that is familiar to next main provider in the chain of care. This ensures that the plan will be familiar to and followed by those who will follow-up in the patient's home.

PACT documents its assessments and proposals for further plans in both municipal and hospital EHR. PACT coordinates and takes responsibility for the sharing the information with all involved actors. PACT also initiates dialogue, arranges transfer meetings as needed, and offers to assist homecare staff the first few days after hospital discharge if needed. PACT preferably hand-off the plan, to the patient's primary contact in the home nursing services, so that responsibility for follow-up is clear. Unfortunately, today, this function is not always in place. PACT will then addresses the unit's management and request that such a responsible person be appointed.

### 9. Allocation of resources

PACT collaborates closely with the homecare service and the municipal allocation authorities regarding the need for further services in the patient's home. PACT has no authority regarding the allocation of municipal services but based on PACTs interdisciplinary assessments, the team's professional evaluation is taken into account by the municipal authorities.

### 10. Interdisciplinary risk analysis, at an individual level

During the process, after necessary information is obtained from the patient, dependents, the patient's significant others and involved healthcare professionals, PACT makes an interdisciplinary risk analysis in each individual patient pathway. This risk analysis is prepared internally in PACT, but preferably, in the future, it will also be possible to include both the patient, dependents and involved actors in the healthcare service.

The risk analysis is based on a "Bowtie model", where single events are analysed, both regarding cause, consequence and timeline. The risk analysis aims to identify the relationships in cause and consequence and what risk mitigating activities that can be introduced.

### 11. Planning

After completing the risk analysis and other necessary information is obtained, PACT's further contribution towards the patient and home care service is clarified. If possible, the patient’s long-term objective and follow-up plans will be set. In close dialogue with the patient, the following questions are clarified:

• What is needed for the patient to reach her goals and objectives?

• Who should be involved?

• What must be organized?

• Do earlier decisions have to be changed?

• Is the necessary competence and expertise in place?

• What is PACT's contribution to the startup?

• How will the transfer to the next level of the healthcare service take place?

• Who is in charge of the follow-up plan in the long term?

PACTs mini-team makes an interdisciplinary summary, which is documented in the patient's EHR in both primary and secondary care, and the summary is also communicated to all involved professionals.

The plan that is completed should be a targeted, proactive plan based on the patient's objectives and risk assessment. A comprehensive plan is made. The treatment and follow up-plan is designed with the patient and in cooperation with actors who are part of the patient's surroundings.

### 12. Implementation of the follow-up plan in the patients home

(see circle in PSHT flow chart)

As early as possible, the role of PACT needs to be clarified when it comes to the implementation of the follow-up plan at home. It is recommended that the homecare service take over the follow-up as soon as possible. The ideal is a good collaboration from the start so that the overlap is seamless and without delay. PACTs experience indicates that there is a large variation in individual homecare service units of how quickly regular healthcare services are ready to take responsibility for their independent follow-up. PACT can assist with initiating assessments and evaluation, and PACT is also available for guidance and transfer of competence if needed. PACT also helps to adjust the healthcare according to needs, in close collaboration with the GP, hospital ward, homecare service and the Municipal allocation authority. PACT's efforts can be summed up with its own variant of the PDCA circle (ref. E. Deming), with the following elements:

1. Start follow-up

2. Supervise / collaborate

3. Evaluate

4. Adjust

### 13. Transfer of follow-up / Exit PACT:

When it is time for PACT to withdraw and transfer responsibility of follow-up to the homecare service, the PACT mini-team meets. The withdrawal is clarified with the patient, significant others, dependents and other involved actors. PACT assesses issues such as: "Is the patient pathway on the right track? Have the patients’ needs and objectives been taken care of? Is the homecare service ready to take over responsibility? Is there any plan for further follow-up and is the plan in use? Transition meetings are conducted if needed.

PACT summarises its assessments and conclusions in an interdisciplinary summary, documented in the patient's EHR that also is sent to the patient and involved actors.


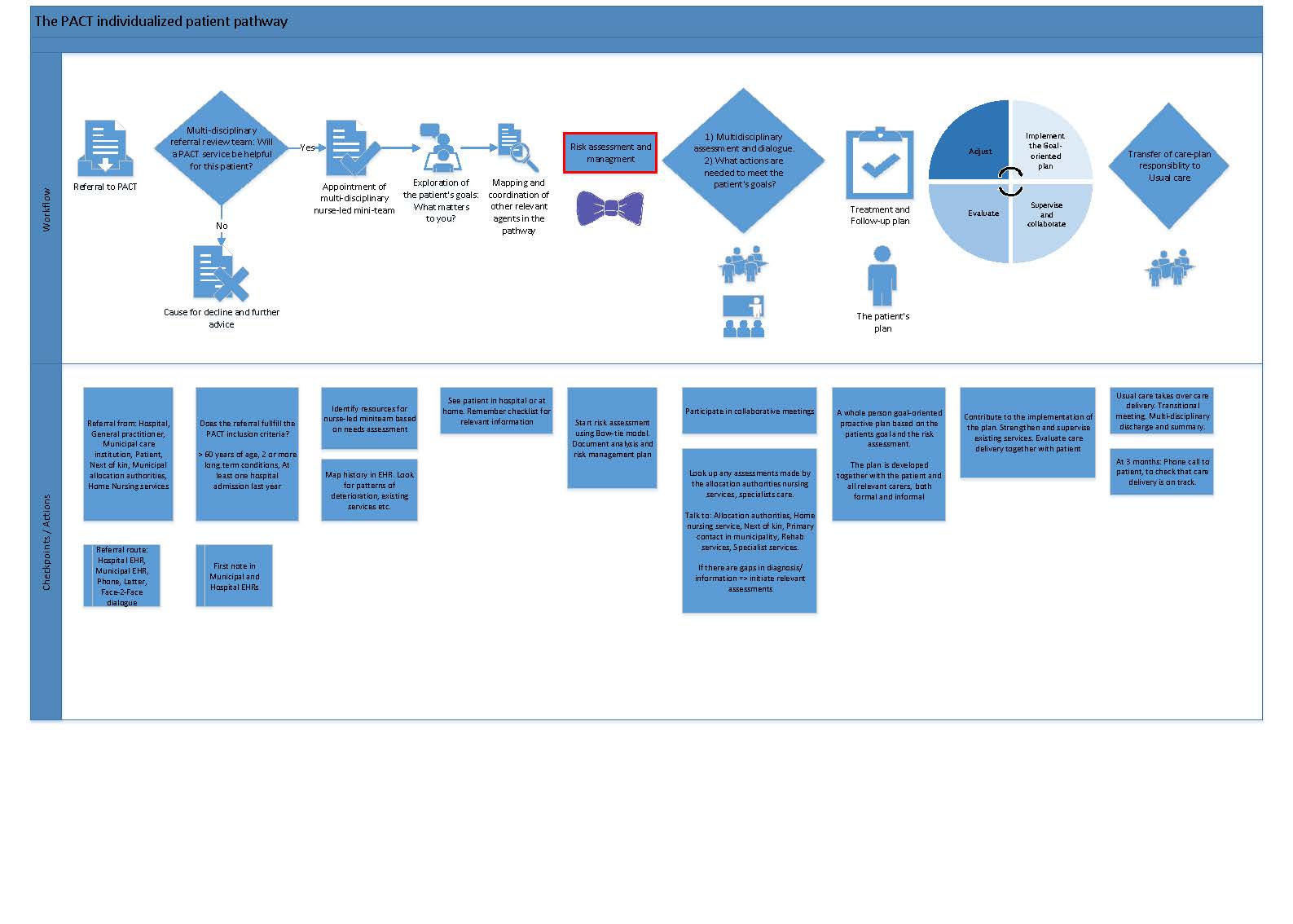

Supplement: Supplementary file 1 — The PACT care model. A stronger and more detailed qualitative description of the PACT intervention. (DOCX 210 kb) [file 12913_2019_4397_MOESM1_ESM.docx]
