# Supplementary material for: Person-centred, integrated and pro-active care for multi-morbid elderly with advanced care needs: a propensity score-matched controlled trial
Source: BMC Health Serv Res. 2019 Oct 3;19:682. doi: 10.1186/s12913-019-4397-2 (PMC6777026; doi:10.1186/s12913-019-4397-2)
Supplement: Supplementary file 2 — Supplementary analyses. This file includes additional tables showing: Crude results for all outcomes. Pooled adjusted results for all outcomes, showing I2 (the heterogeneity index across sites), with outlier-sites first included and then excluded. The Kaplan-Meier plot for mortality, with outlier sites excluded. (DOCX 53 kb) [file 12913_2019_4397_MOESM2_ESM.docx]

# Supplementary analyses, Patient Centered Team (PACT) study, Norway 2014-16.

Table 1: Crude results. Rates, Rate Ratios, Absolute risk difference and Number Needed to Treat by outcome. The Patient-Centred Team (PACT)-study, Norway 2015-16.

| Outcome | N Ctrl | N PACT | Sum Ctrl | Sum Interv | Follow-up days Ctrl | Follow-up days Interv | Rt Ctrl pr PY | Rt Interv pr PY | RR | RR Lower 95% CI | RR Upper 95% Ci | Abs diff | NNT |
| --- | --- | --- | --- | --- | --- | --- | --- | --- | --- | --- | --- | --- | --- |
| Emergency admissions | 779 | 439 | 614 | 340 | 118 709 | 72 577 | 1,89 | 1,71 | 0,9 | 0,79 | 1,03 | -0,18 | -5,62 |
| Sum emergency bed days * | 540 | 316 | 6509 | 5076 | 79 762 | 51 573 | 29,79 | 35,92 | 1,2 | 6,14 | 1,16 | 1,25 | 0,16 |
| 30 day readmissions | 779 | 439 | 276 | 106 | 118 709 | 72 577 | 0,85 | 0,53 | 0,6 | 0,50 | 0,79 | -0,32 | -3,17 |
| Elective outpatient visits | 779 | 439 | 1756 | 3046 | 118 709 | 72 577 | 5,40 | 15,32 | 2,8 | 2,68 | 3,01 | 9,92 | 0,10 |
| Emergency outpatient visits | 779 | 439 | 246 | 204 | 118 709 | 72 577 | 0,76 | 1,03 | 1,4 | 1,13 | 1,63 | 0,27 | 3,71 |
| 0-3 month mortality | 779 | 439 | 146 | 41 | 62 564 | 37 854 | 0,85 | 0,40 | 0,5 | 0,33 | 0,66 | -0,46 | -2,19 |
| 0-6 months mortality | 779 | 439 | 180 | 74 | 118 709 | 72 577 | 0,55 | 0,37 | 0,7 | 0,51 | 0,88 | -0,18 | -5,52 |

*Sum emergency bed days analysis restricted to PACT patients with an index emergency hospitalization and their controls.

**Abbreviations**: N – Number of patients, Ctrl – Controls, Interv-intervention. Rt –Rate (Event/ population time), PY-PersonYear, RR-Rate Ratio (Rt Interv/Rt Control), 95% CI: 95% Confidence interval, Abs diff – Absolute risk difference (Rt Interv – Rt Ctrl), NNT – Number Needed to Treat/Harm (1/ Abs Diff)

Table 2: Multivariate multilevel adjusted Rate Ratios (RR) for outcomes analyses, by outcomes, sub-group. Results given for pooled results for all sites, and for a homogenous dataset, where outlier sites causing heterogeneity are excluded. Negative Binomial regression for health care utilization outcomes and cox regression for mortality. The Patient-Centred Team (PACT)-study, Norway 2014-16. . Emergency bed day includes bed days from index episode, which is why the analysis is restricted to patients recruited in hospital only.

|  |  | | Pooled, All sites | | | | | | | | | | |  | Pooled, Outliers excl. | | | | | | |  |
| --- | --- | --- | --- | --- | --- | --- | --- | --- | --- | --- | --- | --- | --- | --- | --- | --- | --- | --- | --- | --- | --- | --- |
|  |  | |  | | | | | | 95% CI | | | | |  |  | | | | | | |  |
| Outcome/I^2^/Outliers | Sub-group analysis | | N | | IRR | | p | | Lower | | Upper | | |  | N | | | IRR | | p | |  |
| Emergency Admissions  I^2^: 15%^1^,0%^2^  Outliers: M2-Dis | All, crude | | 1218 | | 0,89 | | 0,40 | | 0,67 | | | 1,17 | |  | 1158 | | | 0,92 | | 0,25 | |  |
|  | All, adjusted | | 1218 | | 0,95 | | 0,00 | | 0,94 | | | 0,96 | |  | 1158 | | | 0,95 | | 0,00 | |  |
|  | Only controls surviving Lead days (1) | | 1195 | | 0,95 | | 0,02 | | 0,91 | | | 0,99 | |  | 1137 | | | 0,95 | | 0,04 | |  |
|  |  | |  | |  | |  | |  | | |  | |  |  | | |  | |  | |  |
|  | Only emergency hospitalizations (2) | | 856 | | 0,90 | | 0,02 | | 0,82 | | | 0,98 | |  | 814 | | | 0,90 | | 0,02 | |  |
|  | Combination of 1 and 2 | | 838 | | 0,90 | | 0,03 | | 0,82 | | | 0,99 | |  | 797 | | | 0,90 | | 0,04 | |  |
|  |  | |  | |  | |  | |  | | |  | |  |  | | |  | |  | |  |
| Sum Emergency Bed days  I^2^ – 88%^1^, 9%^2^  Outliers: M1 Dis, M2 dis |  | |  | |  | |  | |  | | |  | |  |  | | |  | |  | |  |
|  |  | |  | |  | |  | |  | | |  | |  |  | | |  | |  | |  |
|  |  | |  | |  | |  | |  | | |  | |  |  | | |  | |  | |  |
|  | Only emerg hospit (2) | | 856 | | 0,62 | | 0,00 | | 0,49 | | | 0,77 | |  | 562 | | | 0,57 | | 0,00 | |  |
|  | Combination of 1 and 2 | | 838 | | 0,68 | | 0,01 | | 0,52 | | | 0,89 | |  | 550 | | | 0,60 | | 0,00 | |  |
|  |  | |  | |  | |  | |  | | |  | |  |  | | |  | |  | |  |
| Readmissions  I^2^: 12%^1^  No outliers | All, crude | | 1218 | | 0,60 | | 0,01 | | 0,40 | | | 0,89 | |  | No heterogeneity detected – no sensitivity analyses performed | | | | | | |  |
|  | All, adjusted | | 1218 | | 0,64 | | 0,00 | | 0,52 | | | 0,78 | |  |  |  |  |  |  |  |  |  |
|  | Only controls surviving Lead days (1) | | 1195 | | 0,63 | | 0,00 | | 0,51 | | | 0,79 | |  |  |  |  |  |  |  |  |  |
|  |  | |  | |  | |  | |  | | |  | |  |  |  |  |  |  |  |  |  |
|  | Only emergency hospitalizations (2) | | 856 | | 0,71 | | 0,21 | | 0,41 | | | 1,22 | |  |  |  |  |  |  |  |  |  |
|  | Combination of 1 and 2 | | 838 | | 0,72 | | 0,23 | | 0,41 | | | 1,24 | |  |  |  |  |  |  |  |  |  |
| Elective Outpatient visits  I^2^: 95%^1^, 9%^2^ Outlier: M1-Dis, M2-Dis | | All, crude | | 1218 | | 2,32 | | 0,00 | | 2,03 | | | 2,66 | | |  | 803 | | 2,21 | | 0,00 | |
|  |  | All, adjusted | | 1218 | | 2,40 | | 0,00 | | 2,21 | | | 2,61 | | |  | 803 | | 2,30 | | 0,00 | |
|  |  | Only controls surviving Lead days (1) | | 1195 | | 2,41 | | 0,00 | | 2,22 | | | 2,62 | | |  | 787 | | 2,31 | | 0,00 | |
|  |  | Only emergency hospitalizations (3) | | 856 | | 2,26 | | 0,00 | | 2,01 | | | 2,54 | | |  | 562 | | 2,12 | | 0,00 | |
|  |  | Combination of 1 and 2 | | 838 | | 2,27 | | 0,00 | | 2,02 | | | 2,55 | | |  | 550 | | 2,13 | | 0,00 | |
| Emergency Outpatient visits  I^2^: 67%^1^, 0%^2^  Outliers: M1-Dis | |  | |  | |  | |  | |  | | |  | | |  |  | |  | |  | |
|  |  | All, crude | | 1218 | | 0,99 | | 0,71 | | 0,95 | | | 1,04 | | |  | 863 | | 1,00 | | 0,99 | |
|  |  | All, adjusted | | 1218 | | 0,82 | | 0,00 | | 0,73 | | | 0,92 | | |  | 863 | | 0,83 | | 0,02 | |
|  |  | Only controls surviving Lead days (1) | | 1195 | | 0,82 | | 0,00 | | 0,76 | | | 0,88 | | |  | 845 | | 0,82 | | 0,00 | |
|  | | Only emergency hospitalizations (3) | | 856 | | 0,89 | | 0,41 | | 0,67 | | | 1,18 | | |  | 604 | | 0,86 | | 0,45 | |
|  | | Combination of 1 and 2 | | 838 | | 0,90 | | 0,46 | | 0,68 | | | 1,20 | | |  | 591 | | 0,87 | | 0,49 | |
| Mortality 0-3 months  I^2^: 58%^1^, 10%^2^  Outlier: M2-Dis | | All, crude | | 1218 | | 0,44 | | 0,00 | | 0,30 | | | 0,65 | | |  | 1158 | | 0,48 | | 0,00 |  |
|  |  | All, adjusted | | 1218 | | 0,38 | | 0,00 | | 0,24 | | | 0,60 | | |  | 1158 | | 0,41 | | 0,00 |  |
|  |  | Only controls surviving Lead days (1) | | 1195 | | 0,46 | | 0,00 | | 0,28 | | | 0,73 | | |  | 1137 | | 0,48 | | 0,00 |  |
|  |  | Only emergency hospitalizations (3) | | 856 | | 0,32 | | 0,00 | | 0,19 | | | 0,55 | | |  | 814 | | 0,35 | | 0,00 |  |
|  | | Combination of 1 and 2 | | 838 | | 0,39 | | 0,00 | | 0,22 | | | 0,70 | | |  | 797 | | 0,41 | | 0,00 |  |
| Mortality 0-6 months  I^2^: 69%^1^, 0%^2^  Outlier: M2-Dis | |  | |  | |  | |  | |  | | |  | | |  |  | |  | |  |  |
|  |  | All, crude | | 1218 | | 0,68 | | 0,01 | | 0,52 | | | 0,89 | | |  | 1158 | | 0,70 | | 0,01 |  |
|  |  | All, adjusted | | 1218 | | 0,53 | | 0,00 | | 0,37 | | | 0,77 | | |  | 1158 | | 0,57 | | 0,00 |  |
|  |  | Only controls surviving Lead days (1) | | 1195 | | 0,60 | | 0,01 | | 0,41 | | | 0,89 | | |  | 1137 | | 0,63 | | 0,02 |  |
|  | | Only emergency hospitalizations (3) | | 856 | | 0,48 | | 0,00 | | 0,30 | | | 0,78 | | |  | 814 | | 0,54 | | 0,01 |  |
|  | | Combination of 1 and 2 | | 838 | | 0,57 | | 0,03 | | 0,34 | | | 0,94 | | |  | 797 | | 0,61 | | 0,06 |  |

Abbreviations: N – Number of patients, RR-Rate Ratio (Rate Interv /Rate Control), p- probability, 95% CI - 95% confidence interval. Excl – Excluded. I2: Heterogeneity index: % of variation explained by differences across sites [69] in pooled results for 1) all sites and 2) homogenous pooled results with outliers excluded.

**Final model adjustment variables**:

Emergency admissions: Fixed effect: Count of emergency admissions last year, Site. Random effect: site, triplet-stratum ID. Sum emergency inpatient days: Fixed effect: Quintile of lead days, Sum emergency bed days last year, Site. Random effect: site, triplet-stratum ID.

Count 30 day Readmissions: Fixed effect: Quintile of lead days, Count re-admissions last year, Site. Random effect: site, triplet-stratum ID. Planned outpatient visits: Fixed effect: Count planned outpatient visits last year, Site. Random effect: site, triplet-stratum ID.

Emergency outpatient visits: Fixed effect: Quintile lead days, Count emergency outpatient visits last year, Site. Random effect: site, triplet-stratum ID

Mortality 0-3 months: Fixed effects: Quintile lead days, Age, Elixhauser score, Site. Random effect: site, triplet-stratum ID.

Mortality 0-6 months: Fixed effects: Quintile lead days, Count readmission last year, Age, Elixhauser score, Site. Random effect: site, triplet-stratum ID.

**Sub-group analyses**: (1) Only controls surviving Lead days: Controls who survived the intervention group’s median lead days in the hospital. Excluded: Controls who died during the first 4-5 days and their matches. (2) Only intervention patients with an index emergency hospital episode. Excluded: Intervention patients with index episode in the municipality or planned hospitalisation and their matches

Figure 1: **Mortality strictest subgroup analysis**. Kaplan-Meier curves, showing the proportion of patients alive by time, and treatment group at six months follow-up. Included: Only patients and controls recruited by an emergency hospitalisation, and Controls have survived a lead period equal to median intervention-lead period. Pooled data across three sites. Outlier site (M2-distant) is excluded. (N=1158) The Patient-Centred Team (PACT)-study, Norway 2015-16.
